# Preliminary Results: Examining Outcomes Associated with Adherence to a Mediterranean Diet compared to a Mediterranean Ketogenic Diet Using the ICAN Program

**DOI:** 10.1002/alz70860_105075

**Published:** 2025-12-23

**Authors:** Julia L Sheffler, Ravinder Nagpal

**Affiliations:** ^1^ Florida State University College of Medicine, Tallahassee, FL, USA; ^2^ Florida State University, Tallahassee, FL, USA

## Abstract

**Background:**

Both Mediterranean (MEDDIET) and Modified Mediterranean Ketogenic diets (MKD) show benefits for addressing multiple mechanisms of Alzheimer's disease and related dementia risk. We herein conducted a pilot randomized clinical trial (RCT) to compare the effects of adherence to these two dietary patterns across multiple systems. Further, we examined the important role of adherence in a real‐world setting using community‐style programs to administer the interventions.

**Method:**

Data were drawn from the first wave of data collection in this pilot RCT. Participants (*N* = 31) included underrepresented older adults (Mean age = 69.71, SD=5.56) from primarily rural areas (71%). Participants were randomly assigned to complete the 10‐week group, “Improving Cognitive Aging through Nutrition” (ICAN) program using either MEDDIET or MKD. Participant biological, cognitive, adherence, and psychosocial outcomes were assessed in‐person at baseline and 10‐weeks. Additional adherence data was collected through weekly surveys reporting on MEDDIET adherence, self‐reported adherence ratings, and at‐home urine ketone testing.

**Result:**

The program supported good adherence to both diets, demonstrating real‐world applications of the findings. Significant differences on multiple clinical measures were identified, including memory, weight loss, blood glucose, cholesterol, and gut metabolites related to adherence to each diet.

**Conclusions:**

An MKD may provide unique benefits for ADRD risk reduction compared to the MEDDIET, and the ICAN program demonstrates feasibility to provide the tools necessary to allow older adults to implement either of these diets into their lifestyle.

